# Supplementary material for: Antibiotic-Loaded Hyperbranched Polyester Embedded into Peptide-Enriched Silk Fibroin for the Treatment of Orthopedic or Dental Infections
Source: Nanomaterials (Basel). 2022 Sep 13;12(18):3182. doi: 10.3390/nano12183182 (PMC9503932; doi:10.3390/nano12183182)
Supplement: Supplementary file 1 [file nanomaterials-12-03182-s001.zip › nanomaterials-1923461-supplementary.pdf]

nanomaterials

Supplementary Materials

Antibiotic-Loaded Hyperbranched Polyester Embedded into Peptide-Enriched Silk Fibroin for the Treatment of Orthopedic or Dental Infections

Zili Sideratou [1,$], Marco Biagiotti [2,$], Dimitris Tsiourvas [1,$], Katerina N. Panagiotaki [1], Marta V. Zucca [2], Giuliano Freddi [2], Arianna B. Lovati [3,§] and Marta Bottagisio [4,§,*]

[1] **Institute** of Nanoscience and Nanotechnology, NCSR "Demokritos", 15310 Aghia Paraskevi, Greece;
[2] Silk Biomaterials srl, Via Cavour 2, 22074 Lomazzo, Italy
[3] IRCCS Istituto Ortopedico Galeazzi, Cell and Tissue Engineering Laboratory, Via R. Galeazzi 4, 20161, Milan, Italy
[4] IRCCS Istituto Ortopedico Galeazzi, Laboratory of Clinical Chemistry and Microbiology, Via R. Galeazzi 4, 20161, Milan, Italy
$ and § equally contributed
* Correspondence: marta.bottagisio@grupposandonato.it; tel. +390266214069

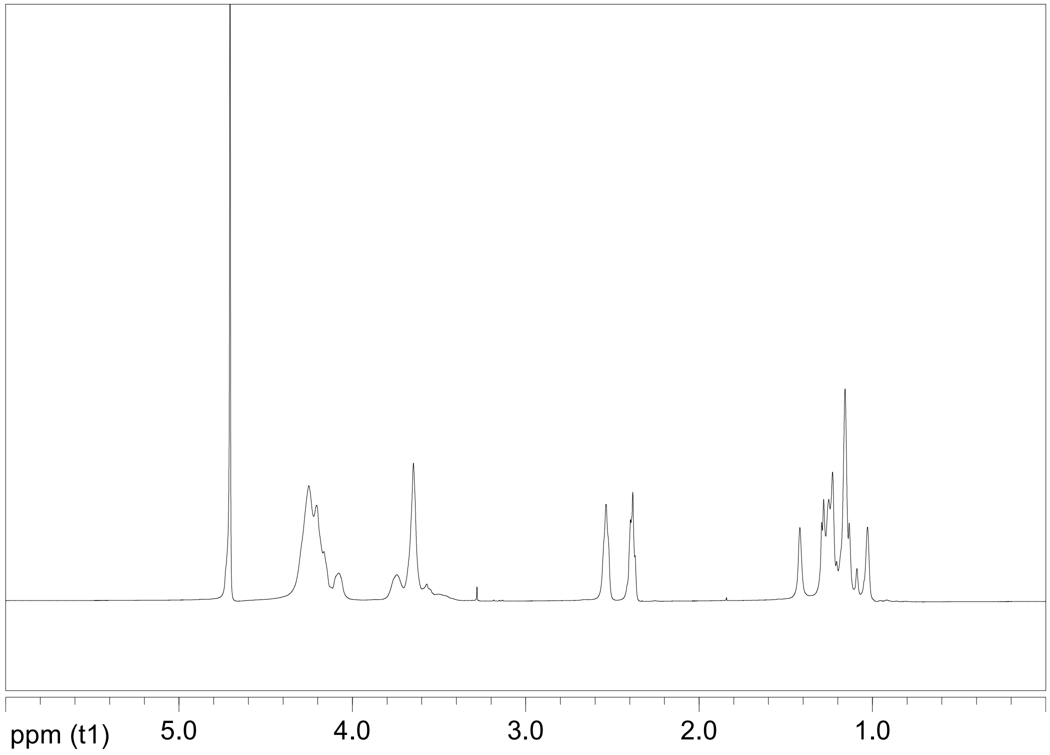

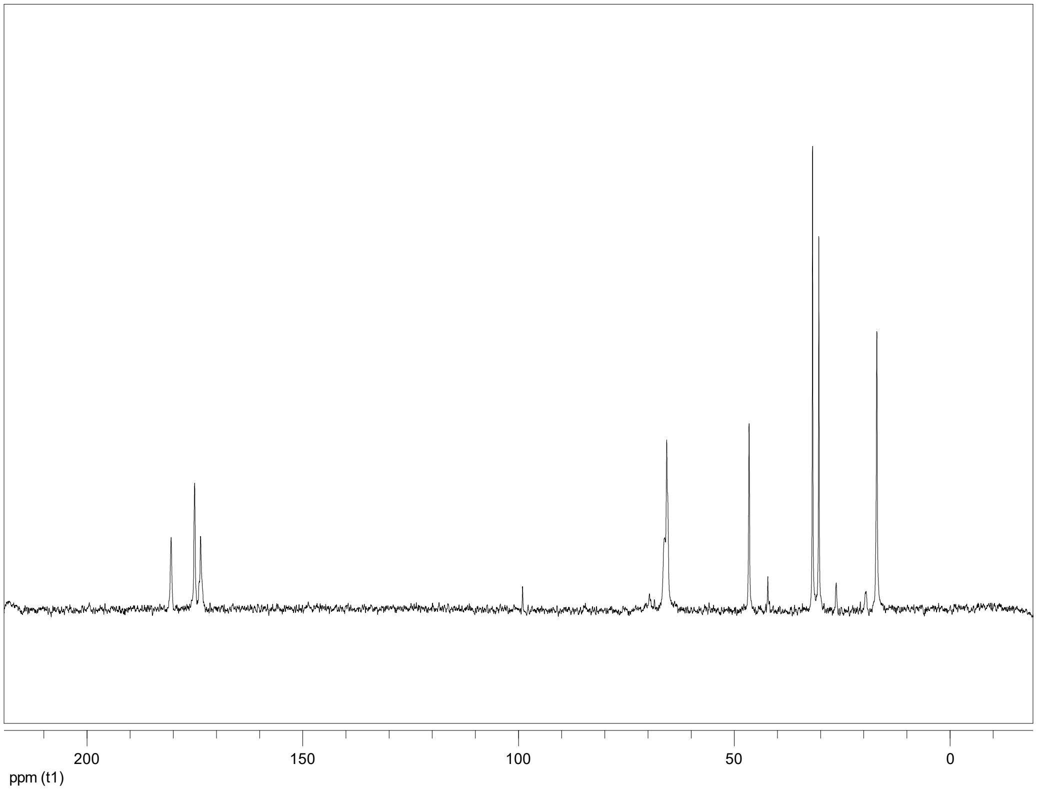

Figure S1. ^{1}H NMR and ^{13}C NMR spectra of CHAP in D_2O.

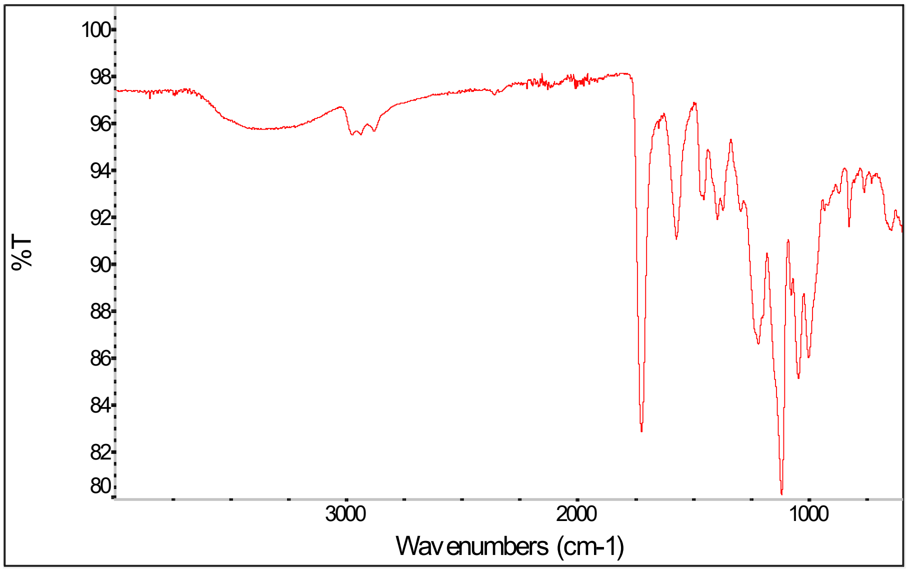

Figure S2. FTIR spectrum of CHAP.

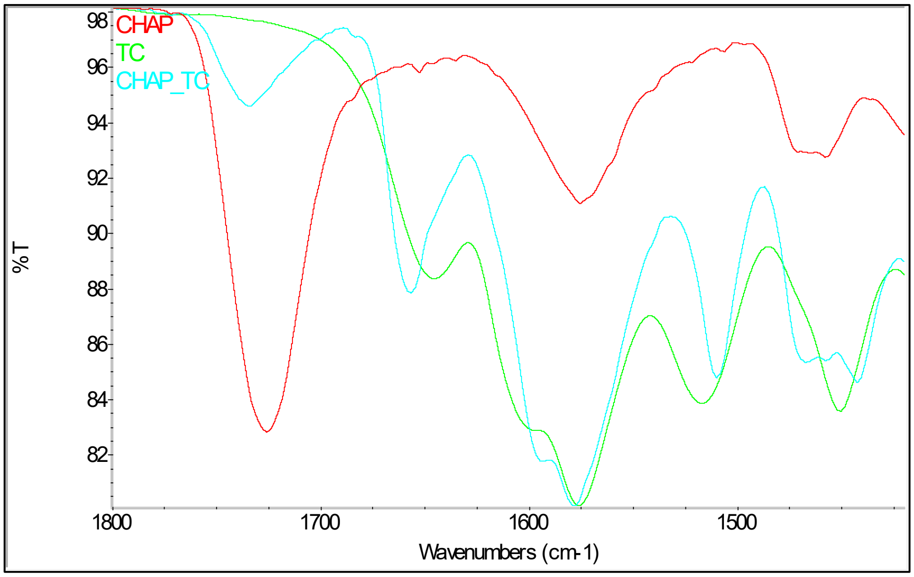

Figure S3. FTIR spectra of CHAP, tetracycline (TC) and TC-loaded CHAP nanoparticles (CHAP_TC).

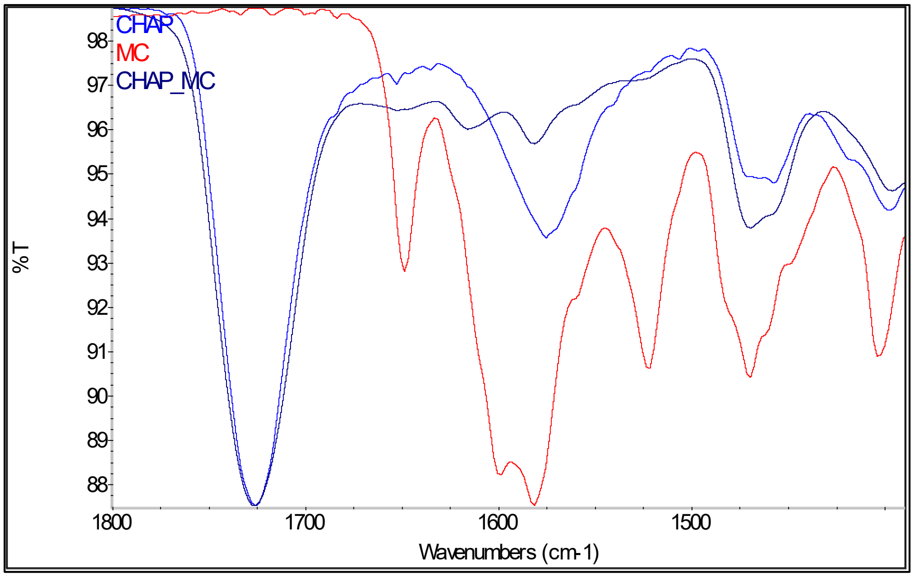

Figure S4. FTIR spectra of CHAP, minocycline (MC) and MC-loaded CHAP nanoparticles (CHAP_MC).

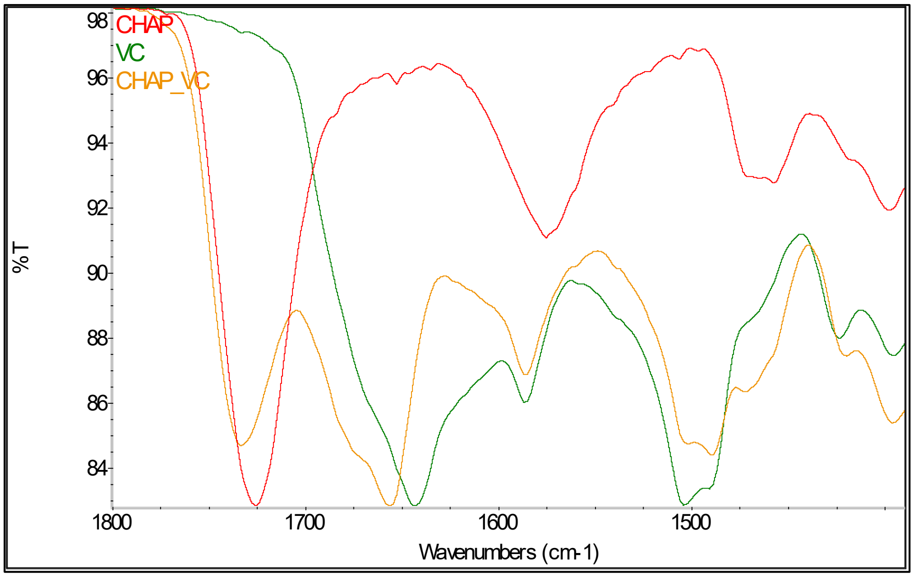

Figure S5. FTIR spectra of CHAP, vancomycin (VC) and VC-loaded CHAP nanoparticles (CHAP_VC).

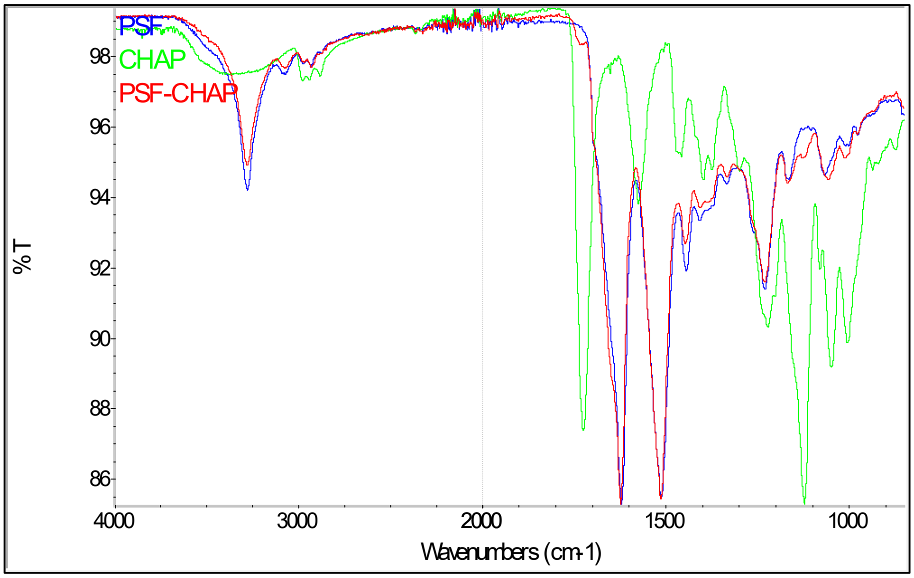

Figure S6. FTIR spectra of CHAP, PSF and PSF@CHAP20 sponges.
